# Prevalence of central sensitization and somatization in adults with temporomandibular disorders—a prospective observational study

**DOI:** 10.22514/jofph.2024.037

**Published:** 2024-12-12

**Authors:** Piotr Seweryn, Marta Waliszewska-Prosol, Marcin Straburzynski, Joanna Smardz, Sylwia Orzeszek, Wojciech Bombala, Marta Bort, Andrej Jenca Jr, Anna Paradowska-Stolarz, Mieszko Wieckiewicz

**Affiliations:** ^1^Department of Experimental Dentistry, Wroclaw Medical University, 50-425 Wroclaw, Poland; ^2^Department of Neurology, Wroclaw Medical University, 50-556 Wroclaw, Poland; ^3^Department of Family Medicine and Infectious Diseases, University of Warmia and Mazury, 10-719 Olsztyn, Poland; ^4^Statistical Analysis Center, Wroclaw Medical University, 50-368 Wroclaw, Poland; ^5^Department of Stomatology and Maxillofacial Surgery, Faculty of Medicine, Pavol Jozef Safarik University in Kosice and Akademia Kosice, 040 01 Kosice, Slovakia; ^6^Department of Maxillofacial Orthopedics and Orthodontics, Wroclaw Medical University, 50-425 Wroclaw, Poland

**Keywords:** Temporomandibular disorders, Masticatory muscle pain, Orofacial pain, Central Sensitization, Somatization

## Abstract

Temporomandibular disorders (TMD) comprise a group of conditions affecting the masticatory muscles, the temporomandibular joints and associated structures, often manifesting as orofacial pain and functional limitations of the mandible. Central sensitization (CS) is gaining increasing attention in research focused on pain syndromes and somatization, playing a significant role in the pain experience. This study investigates the prevalence of CS and somatization among TMD patients, analyzing their relationships with TMD diagnoses and the intensity of chronic masticatory muscle pain (MMP). A prospective observational study was conducted with 214 adult participants diagnosed with TMD, based on the Diagnostic Criteria for Temporomandibular Disorders (DC/TMD). The Central Sensitization Inventory (CSI) and the Somatic Symptom Scale-8 (SSS-8) were utilized to assess CS and the burden of somatic symptoms, respectively. Furthermore, the patients were assessed for MMP, and the average pain in these muscles was calculated. Statistical analysis investigated correlations between CSI and SSS-8 scores, specific TMD diagnoses and MMP intensity. Most participants did not surpass the subclinical level for CS as assessed by the CSI. Women reported higher SSS-8 scores than men, suggesting sex differences in somatic symptom reporting. No significant relationship was found between specific TMD diagnoses and levels of CS or the SSS-8. However, a significant correlation was observed between SSS-8 scores and the intensity of chronic MMP, underscoring the impact of the intensity of chronic MMP on the perception of somatic symptoms among TMD patients. Additionally, the group with subclinical levels of CS presented significantly lower SSS-8 scores than other groups. This study highlights a lower-than-expected prevalence of CS among TMD patients. Higher levels of somatization were related to higher levels of CS and greater MMP. The findings suggest that TMD management should not only address specific pain sources but also consider the broader psychosocial aspects of the disorders, especially in chronic types.

## 1. Introduction

Temporomandibular disorders (TMD) are a group of disorders primarily affecting the masticatory muscles, temporomandibular joints and related structures [1]. They can manifest as pain in the orofacial region and/or functional limitations such as limited mouth opening or a clicking sound during mandible movement [1, 2]. TMD impacts a considerable portion of the population, with some studies suggesting that approximately 5–12% of the population is affected [3]. A recent study on a Polish cohort reported over 55% of adult participants presented with at least one of the symptoms [4]. It is also considered the second most common cause of orofacial pain and the second leading cause of pain and disability in the musculoskeletal system, right after low-back pain [5, 6]. The occurrence is twice as common in women as in men [7]. These disorders frequently evolve into chronic or recurrent symptoms, which can diminish the quality of life, impacting both physical and emotional health, as well as overall functional capability [8, 9, 10].

The origins of TMD are complex, multilayered and often not easily identified [11, 12]. The biopsychosocial model tends to be the most comprehensive framework for understanding and managing these conditions [13]. This model recognizes that TMD are not solely a physical health issue but is influenced by a complex interplay of biological, psychological and social factors [13, 14].

Despite the mentioned model, TMD’s underlying pathophysiology remains insufficiently understood, which underlines the necessity for ongoing research, particularly into the neurobiological mechanisms contributing to TMD pain and its persistence [11, 15, 16].

In this context, a growing interest in the central mechanisms of pain can be observed in the literature [1, 16]. Central sensitization (CS) has gained increasing focus due to its possible contribution to the persistence and severity of pain [1]. This phenomenon can be understood as an amplified response of the central nervous system to sensory stimuli and peripheral nociception [17]. CS is characterized by hyperexcitability in the dorsal horn neurons in the spinal cord, which ascend along the spinothalamic tract [18]. In the context of orofacial pain, hyperexcitability of second-order neurons in the trigeminal nucleus caudalis (TNC) can lead to CS [19]. It can be indicated by a heightened and sustained response to painful stimuli (hyperalgesia) and the experience of pain in response to nonpainful stimuli (allodynia) [18].

The precise mechanisms provoking central sensitization remain unclear, indicating the need for further research [1]. Nevertheless, it is hypothesized that CS may be associated with enhancement in the synaptic response to a given input, either by increased neurotransmitter release or heightened receptor sensitivity. This condition is often a result of prolonged pain exposure [19]. Another possible cause is the reduction or loss of inhibitory controls, leading to heightened pain sensitivity [20].

TMD can be considered one of the central sensitization syndromes (CSS) [21]. Moreover, it is often associated with other CSS [22]. In TMD patients, CS results in a reduced pressure pain threshold (PPT) and can lead to the sensation of pain despite the absence of tissue damage [23]. Additionally, it emerges as an important aspect of the pathophysiology of different types of musculoskeletal pain, including muscle-type TMD [18]. Therefore, CS requires further research to fully understand its implications in orofacial pain.

Considering CS as a relevant factor in the pathophysiology of TMD and its influence on pain perception, it becomes reasonable to assess the broader range of somatic symptoms experienced by patients. Somatization refers to the experience and reporting of physical symptoms that cannot be fully explained by any underlying medical condition but are linked to psychological factors [24]. Given that individuals with TMD often experience such ailments, assessing this parameter is important for a comprehensive evaluation of their condition [25]. Taking into account that CS is also associated with increased sensitivity to pain and the experience of pain even in the absence of a structural cause, it has much in common with somatization, and they may have a similar underlying cause [26]. Addressing somatization is essential as it can exacerbate the patient’s overall symptom burden [24]. Incorporating analysis of somatization allows for better identification of patients who may benefit from interventions targeting both the physical and psychological aspects of TMD. This holistic approach can lead to more targeted treatment and better outcomes [27]. In this regard, the Somatic Symptoms Scale-8 (SSS-8) is proving to be an appropriate tool [28]. This questionnaire, by measuring the overall burden of bodily symptoms, will allow for the analysis of how much CS is affecting patients’ symptoms. Assessing and analyzing this parameter might have a significant impact on understanding the etiology of TMD, as its symptoms often overlap with other conditions that are also impacted by CS [29]. This brief questionnaire could be easily implemented in clinical practice due to its simplicity and ease of completion.

The primary aim of this study was to investigate CS using the Central Sensitization Inventory (CSI) questionnaire and to assess the relationship between these findings and the outcomes of SSS-8 and TMD diagnoses. Furthermore, the secondary aim was to examine the relationship between the intensity of chronic masticatory muscle pain and the results obtained from both questionnaires.

The authors hypothesized that individuals with TMD exhibit high levels of central sensitization as measured by the CSI. Furthermore, it was anticipated that these high levels of CSI scores correlate positively with elevated scores of the SSS-8. Additionally, authors hypothesize that the scores indicated by both the CSI and SSS-8 questionnaires were associated with the intensity of masticatory muscle pain.

## 2. Material and methods

The details regarding study design are presented on Fig. 1.

**Fig. 1.** Flowchart of the study design.

### 2.1 Study design

This prospective observational study enrolled 214 adult participants seeking treatment at the Outpatient Clinic for Temporomandibular Disorders at the University Dental Center in Wroclaw, Poland.

### 2.2 Participants

The study involved 214 adult Caucasian patients with TMD, comprising 160 women (75%) and 54 men (25%). The mean age of the participants was 37.92 (±14.14) with an age range of 18–73. The patients were categorized into the following age groups: 112 individuals aged 18–35 years, 69 individuals aged 36–55 years and 32 individuals aged over 56 years. With a female-to-male ratio of 3:1, women dominated the sample group. In the women’s group, the mean age was 38.53 (±14.59), while in the men’s group it was 36.09 (±12.66). This difference was not statistically significant (*p* = 0.273).

### 2.3 Inclusion and exclusion criteria

The inclusion criteria for the study group were as follows: (1) age of 18 years or above, (2) informed consent to participate in the study, (3) diagnosis of TMD based on the Diagnostic Criteria for Temporomandibular Disorders (DC/TMD) examination [5], and (4) presence of chronic primary and secondary masticatory muscle pain (MMP) by the International Classification of Orofacial Pain, 1st edition (ICOP) criteria for chronic myofascial orofacial pain diagnosis such as chronic primary myofascial orofacial pain or chronic frequent primary myofascial orofacial pain with pain referral [30]. The exclusion criteria were established as follows: (1) chronic use (over 6 months) of medications affecting neuromuscular function or the central nervous system (CNS), such as analgesics (paracetamol, nonsteroidal anti-inflammatory drugs (NSAIDs), opioids), psychostimulants, antiepileptic drugs, sedatives, myorelaxants, steroids and immunosuppressants, (2) severe neurological, psychiatric, cognitive, systemic and/or autoimmune disorders, (3) alcohol and/or drug addiction, (4) cancer, (5) pregnancy and lactation, (6) presence of any other severe pain condition, (7) presence of any primary and secondary headaches, (8) significant abnormalities in a brain imaging examination (Computed Tomography or Magnetic Resonance Imaging).

### 2.4 Data collection

Patients data were acquired from June 2021 to February 2023. Initially, patients were requested to fill out a series of CSI, SSS-8 and Graded Chronic Pain Scale (GCPS) questionnaires, followed by a comprehensive clinical examination by the DC/TMD guidelines and the ICOP criteria for chronic myofascial orofacial pain. These examinations were performed by a dentists with over than 5 years’ experience in TMD management.

### 2.5 Clinical examination

After completing the questionnaires, all participants underwent a thorough clinical examination. This examination was conducted by experienced dentists (with at least 5 years of clinical practice in temporomandibular disorders and orofacial pain management). They were trained and calibrated according to the protocols provided by the International Network for Orofacial Pain and Related Disorders Methodology [31]. Their training was supervised by a clinician with 10 years of experience in managing TMD and orofacial pain. Diagnoses were established using the Diagnostic Criteria for Temporomandibular Disorders protocol and ICOP guidelines for chronic myofascial orofacial pain [30, 32]. Each side was evaluated independently, allowing for multiple diagnoses on one side. All diagnoses were made according to the taxonomy of DC/TMD [33]. During the clinical examination, each patient was requested to provide a rating for the perceived pain experienced during the palpation of the masseter and temporal muscles on each side individually. Subsequently, the average of these ratings was calculated. The numeric rating scale (NRS), a widely utilized and well-validated tool for measuring pain intensity, was employed for this purpose. The scale ranges from 0 to 10, with 0 indicating no pain and 10 representing the worst imaginable pain [34].

### 2.6 Questionnaires

#### 2.6.1 Central sensitization inventory (CSI)

The evaluation of CS was conducted using the CSI, a valid and reliable tool for screening CS (Cronbach’s alpha = 0.92, test-retest reliability = 0.082) [35]. For this study, a validated and culturally adapted version of the CSI questionnaire was employed [36, 37]. This self-reported instrument consists of two parts. Part A includes 25 questions about health-related symptoms typically observed in Central Sensitivity Syndromes (CSS), such as emotional distress, past traumas, headaches or jaw pain. Patients score each answer on a scale from 0 (never) to 4 (always). The highest possible score is 100, with a score of 40 considered the cut-off point [38]. This questionnaire categorizes patients into the following groups: subclinical (0–29), mild (30–39), moderate (40–49), severe (50–59) and extreme (60–100). Part B of the questionnaire includes questions about CS-related disorders, such as Restless Leg Syndrome, Chronic Fatigue Syndrome, or Fibromyalgia [35]. This part is unscored and does not contribute to the overall scoring of the CSI [35].

#### 2.6.2 Somatic symptom scale-8 (SSS-8)

SSS-8 is a self-reported tool for measuring the burden of somatic symptoms. It is an abbreviated version of the well-validated Patient Health Questionnaire-15 (PHQ-15) [39]. The SSS-8 was designed to evaluate the severity of illness in patients with somatic symptoms and related disorders (SSRD) [39]. It has demonstrated good reliability and internal consistency, proving its usefulness for research purposes (Cronbach’s alpha = 0.72) [40]. The SSS-8 consists of eight questions regarding somatic symptoms such as sleep disturbances, dizziness or headache. Each item is scored using a 5-point Likert scale (0–4), where “0” indicates “not at all” and “4” indicates “very much”. The results can be classified into five categories: “no to minimal” symptoms represented by 0–3 points, “low” symptoms by 4–7 points, “medium” symptoms by 8–11 points, “high” symptoms by 12–15 points and “very high” symptoms by a score of 16–32 [39].

#### 2.6.3 Graded chronic pain scale (GCPS)

The GCPS is an integral component of the Axis II screeners of DC/TMD protocol [5]. This scale encompasses six questions assessing pain over the preceding 3–6 months, with responses rated on a 0–10 scale. A cumulative score of 50 out of 100 or higher indicates the high intensity of pain [41]. An additional seventh question evaluates the number of days the patient has been unable to engage in routine activities due to pain. The overall score is derived from three subscales: characteristic pain intensity score, disability score and disability points score [5]. These scores categorize patients into one of five pain severity grades: Grade 0 indicates no pain, Grade 1 represents low disability and low intensity, Grade 2 corresponds to low disability but high intensity, Grade 3 signifies high disability with moderate limitation, and Grade 4 denotes high disability with severe limitation [41].

### 2.7 Statistical analysis

For statistical analysis of quantitative data in independent groups, the Mann-Whitney U test was used for two groups, and for more than two groups, the Kruskal-Wallis test with Dunn’s *post-hoc* test was applied to data that did not comply with a normal distribution. To ensure adequate power for the Kruskal-Wallis test, the calculations were based on an expected effect size of 0.25, with a power of 0.8 and an alpha level of 0.05, resulting in a total sample size requirement of 200 subjects across a maximum of five groups. The Spearman Correlation coefficient was used to demonstrate the relationship between the quantitative variables. For this analysis, the expected Spearman correlation coefficient was set at 0.15 with a standard deviation of 0.05. The power analysis indicated that a minimum sample size of 155 was necessary to achieve a power of 0.8 at the 0.05 alpha level. For statistical analysis of categorical data, the Chi-square test was applied. Power calculations for the Chi-square test, considering a maximum table size of 3 × 5 and an expected medium effect size of 0.3, suggested a total sample size of 167 to achieve the desired power of 0.8 and an alpha of 0.05. A *post-hoc* test (the chisq.*post.hoc* function from the fifer 1.0 package in R) was used when necessary. A statistically significant difference was accepted at *p* < 0.05. The analyses were carried out using TIBCO Software Inc. (2017) Statistica (data analysis software system, version 13, Palo Alto, CA, USA) and the R environment.

For statistical analysis depending on the type of diagnosis, patients were divided into the following categories: (1) temporomandibular joint disorders (n = 27, women = 18, men = 9), (2) masticatory muscle disorders (n = 74, women = 56, men = 18), and (3) comorbid masticatory muscle and temporomandibular joint disorders (n = 107, women = 82, men = 25).

## 3. Results

### 3.1 Temporomandibular disorders distribution

The following diagnoses of temporomandibular joint disorders were identified in the study group: disc displacement with reduction, disc displacement without reduction, arthralgia, degenerative joint disease and subluxation. Regarding masticatory muscle disorder diagnoses, myalgia, myofascial pain and myofascial pain with referral were established. Among the mentioned diagnoses, myalgia was the most common, affecting 161 individuals (75%). The second most frequent diagnosis within the study group was disc displacement with reduction, occurring in 78 participants (36%), and the third most common diagnosis was arthralgia, observed in 41 individuals (19%). The fourth most prevalent disorder was myofascial pain with referral, identified in 19 participants (9%).

To analyze the correlation between gender and the type of TMD diagnosis, a chi-square test was conducted, but it showed no statistically significant difference (χ^2^ (2) = 1.1707, *p* = 0.557). To assess the difference in age between diagnosis-type groups, a Kruskal-Wallis test was conducted, which also showed no statistically significant difference (*p* = 0.8324). In the assessment of the relationship between gender and pain intensity, the Kruskal-Wallis test was utilized again. The analysis demonstrated that women presented statistically significantly higher scores in all measured parameters: the left masseter muscle (*p* = 0.001), right masseter muscle (*p* < 0.001), left temporal muscle (*p* = 0.019) and right temporal muscle (*p* = 0.034) (Table 1).

**Table 1.** Sex-based distribution of pain severity in masticatory muscles.

	Left masseter muscle		Right masseter muscle		Left temporal muscle		Right temporal muscle	
	Female	Male	Female	Male	Female	Male	Female	Male
Mean	4.944	3.389	5.356	3.778	3.656	2.500	3.781	2.741
95% CI Mean Upper	5.428	4.132	5.831	4.498	4.167	3.211	4.284	3.472
95% CI Mean Lower	4.460	2.646	4.881	3.057	3.145	1.789	3.279	2.010
Std. Deviation	3.098	2.722	3.043	2.640	3.274	2.604	3.219	2.679

*CI: Confidence Interval.*

### 3.2 Temporomandibular disorders, central sensitization inventory (CSI) and graded chronic pain scale (GCPS)

In the CSI questionnaire, 139 participants (65%) obtained a score that classified them into the “subclinical” category. Thirty-five subjects (16%) were categorized into the “mild” group, 21 (10%) achieved the “moderate” category, “severe” was obtained by 15 participants (7%) and 4 participants (2%) were assigned to the “extreme” group (Table 2).

**Table 2.** Group divisions by sex in the central sensitization inventory.

		Women	Men	Total
Subclinical		93	46	139
	% of gender group	58.13%	85.19%	
	% of study group	43.46%	21.5%	64.95%
Mild		29	6	35
	% of gender group	18.13%	11.11%	
	% of study group	13.55%	2.8%	16.36%
Moderate		21	0	21
	% of gender group	13.13%	0	
	% of study group	9.81%	0	9.81%
Severe		13	2	15
	% of gender group	8.13%	3.7%	
	% of study group	6.07%	0.93%	7.01%
Extreme		4	0	4
	% of gender group	2.50%	0	
	% of study group	1.87%	0	1.87%

Regarding the relationship between age and the results of the CSI, the Kruskal-Wallis analysis did not reveal any statistically significant differences between the groups (*H* (4, 214) = 7.861, *p* = 0.096). However, significant differences between women and men were observed (*p* < 0.005), with a higher percentage of men achieving the “subclinical” category. Detailed information about gender distribution can be found in Tables 2 and 3.

**Table 3.** Descriptive statistics of central sensitization inventory.

Descriptive statistics	CSI	
	Female	Male
Number	160	54
Mean	0.787	0.222
SD	1.107	0.634
Minimum	0.000	0.000
Maximum	4.000	3.000

*SD: Standard Deviation; CSI: Central Sensitization Inventory.*

To investigate the relationship between the type of TMD diagnosis and the level of the CSI, a chi-square test was performed, followed by *post-hoc* analyses. These analyses did not reveal any statistically significant associations (*p* = 0.5617). To further clarify the data, the relationship was also examined by comparing the “subclinical” group with the remaining groups combined. Again chi-squared analysis was performed. No statistically significant differences were found between the groups both for muscle type diagnosis (*p* = 0.07) and joint type diagnosis (*p* = 0.776).

When it comes to pain intensity of the masseter and temporal muscles, several associations with the CSI were observed. The “subclinical” group significantly differed from the “moderate” group in the pain intensity of the right masseter muscle (*p* = 0.012). Regarding the temporal muscles, the “extreme” group differed significantly from the “subclinical” group for both the left (*p* = 0.013) and right (*p* = 0.027) temporal muscles. In all mentioned cases, the “subclinical” group exhibited lower pain intensity. No statistically significant differences were observed for the remaining groups (*p* > 0.05). Furthermore, masticatory muscle pain was analyzed by comparing the “subclinical” group with all other CSI groups combined. This analysis showed significantly lower scores in the “subclinical” group for the left masseter muscle (*p* = 0.003), right masseter muscle (*p* < 0.001), left temporal muscle (*p* = 0.014) and right temporal muscle (*p* < 0.001). Detailed information about group descriptives can be found in the Table 4.

**Table 4.** Group descriptives for masticatory muscle pain analysis between subclinical group and remaining groups combined.

Group		N	Mean	SD	SE	Coefficient of variation
Left Masseter muscle						
	Subclinical	139	4.094	3.017	0.256	0.737
	Remaining groups combined	75	5.400	3.027	0.350	0.561
Right Masseter muscle						
	Subclinical	139	4.374	3.003	0.255	0.687
	Remaining groups combined	75	6.040	2.758	0.318	0.457
Left temporal muscle						
	Subclinical	139	2.914	2.858	0.242	0.981
	Remaining groups combined	75	4.200	3.507	0.405	0.835
Right temporal muscle						
	Subclinical	139	2.971	2.899	0.246	0.976
	Remaining groups combined	75	4.533	3.273	0.378	0.722

*N: number of participants; SD: Standard Deviation; SE: Standard Error.*

Additionally, an analysis was conducted to examine the relationship between GCPS and CSI scores. The results were again analyzed by comparing the “subclinical” group with the remaining groups combined, using the Mann-Whitney U test. The analysis revealed statistically significantly lower GCPS scores in the “subclinical” group (*p* = 0.001). Next, a sex-based analysis was performed. In the group of men, the difference between the “subclinical” group and the remaining groups combined was not statistically significant, although it nearly reached the level of significance (*p* = 0.052). In the women’s group, the difference between the subclinical group and the remaining groups combined was statistically significant (*p* < 0.001), with considerably lower GCPS scores observed in the subclinical group.

### 3.3 Temporomandibular disorders, somatic symptom scale-8 (SSS-8) and graded chronic pain scale (GCPS)

In the context of the SSS-8, the distribution of participants across the various groups was as follows: 53 individuals (25%) were assigned to the “no to minimal” symptom category, 51 participants (24%) to the “low symptoms” group, 47 individuals (22%) to the “medium” symptom level, 33 participants (15%) to the “high” symptom category, and 30 individuals (14%) to the “very high” symptoms group.

For analysis of the relationship between SSS-8 scores and age, Spearman’s rank correlation was employed. The result was statistically significant with a correlation coefficient = 0.173, indicating only a slight linear relationship between the variables. This outcome suggests that there is a minor tendency for SSS-8 scores to increase with age.

In terms of the relationship between SSS-8 scores and gender, the Mann-Whitney U test was used. It revealed a significant difference between groups, with women exhibiting significantly higher scores (*p* = 0.018) (Fig. 2).

**Fig. 2.** Somatic symptom scale-8 (SSS-8) results divided by gender.

To analyze the relationship between the type of TMD diagnosis and the SSS-8 severity level, the Kruskal-Wallis test was conducted. The result was not statistically significant (*p* = 0.553), indicating no significant differences in median SSS-8 scores across different TMD diagnosis groups.

The pain levels and their correlation with SSS-8 scores were examined using Spearman’s rank-order correlation test. Significant results were obtained for three of the four tested masticatory muscles. The values of Spearman’s rank order are as follows: left masseter muscle (*r*_s_ = 0.18, *p* = 0.008), right masseter muscle (*r*_s_ = 0.12, *p* = 0.059), left temporal muscle (*r*_s_ = 0.18, *p* = 0.005) and right temporal muscle (*r*_s_ = 0.17, *p* = 0.01). These results demonstrate a weak positive relationship between masticatory muscle pain and SSS-8 scores, suggesting that as masticatory muscle pain increases, there is a slight but statistically significant tendency for SSS-8 scores to increase as well (Fig. 3).

**Fig. 3.** Correlations of results of MMP and SSS-8. MMP: Masticatory Muscle Pain; SSS-8: Somatic Symptom Scale-8; NRS: Numeric Rating Scale.

The Spearman’s rank correlation analysis revealed a statistically significant correlation between GCPS and SSS-8 scores (Spearman’s Rho = 0.308, *p* < 0.001), indicating weak positive correlation between these variables. This suggests that as GCPS scores increase, SSS-8 scores also tend to increase, although the relationship is not very strong.

### 3.4 Cental sensitization inventory (CSI) and somatic symptom scale-8 (SSS-8)

For analyzing the relationship between CSI and SSS-8, Kruskal-Wallis ANOVA was employed. The analysis revealed significant differences between the “subclinical” group and each of the other groups individually (*p* < 0.001), with the “subclinical” group presenting significantly lower SSS-8 scores. The visual representation of the data is presented in Fig. 4. Although the box plots are sequentially arranged with higher values, it cannot be concluded that a higher CSI level corresponds to a greater SSS-8 score. Additionally, significant differences in the sizes of the groups make the results of this test not entirely reliable.

**Fig. 4.** Distribution of SSS-8 results in CSI groups. SSS-8: Somatic Symptom Scale-8; CSI: Central Sensitization Inventory.

To ensure the transparency of our results, an additional analysis was performed by comparing subclinical group and remaining groups combined. The Mann-Whitney U test demonstrated significant differences (*p* = 0.001), with “subclinical group” presenting significantly lower scores of SSS-8 questionnaire.

## 4. Discussion

The findings of this study provided insightful information about CS and somatization and contributed to a better understanding of these concepts, thereby expanding our knowledge of them. The results allow us to assess the relationship between CS, somatic symptom burden and masticatory muscle pain.

The majority of participants were female, which aligns with other studies in this area [9, 10, 42]. This ratio may be attributed to hormonal changes, biological variations or greater pain sensitivity [9]. As in other research papers, myalgia was the most common diagnosis in our study as well [9, 12, 16].

This study employed an extensive list of exclusion criteria aimed at eliminating factors that could interfere with the depiction of central sensitization. Long-term use of medications affecting the neuromuscular system or central nervous system (CNS), such as muscle relaxants or opioids, can alter pain perception [43, 44]. Additionally, this may be associated with cognitive impairments [45]. Severe systemic, neurological or autoimmune diseases necessitate medications that can disrupt nervous system function [46]. Furthermore, alcohol or drug addiction can lead to neurotoxicity, contributing to neuroinflammation and CNS damage, significantly influencing the study’s results [47]. Participants with other severe pain conditions were excluded to focus specifically on central sensitization in patients with temporomandibular disorders, rather than central sensitization in general. Similarly, primary and secondary headaches were excluded, as they require separate investigation.

During this study, the DC/TMD was utilized. It is recognized as the gold standard for diagnosing temporomandibular disorders. It was used to provide a comprehensive framework for TMD identification and classification [48]. This allowed for precise classification of various TMD types, enhanced diagnostic reliability and validity and enabled comparisons with other studies. Additionally, it helped identify specific subgroups within the TMD population and understand the relationship between TMD and related conditions, such as central sensitization, using a standardized diagnostic framework. Interestingly, a majority of TMD patients did not surpass the threshold of the subclinical level for CS in the CSI. This may suggest that CS, as measured by the CSI, is not as prevalent in TMD patients as previously presumed [49]. On the other hand, La Touche *et al.* [49], in their systematic review and meta-analysis, found a significant amount of CSSs among patients with TMD. Cayrol *et al.* [15] suggested that features of CS, such as mechanical hyperalgesia, are more common in patients with chronic TMD. The discrepancy between these results and our study may arise from several reasons. In the present study, the precise duration of the TMD was not specified. Taking into account that CS is generally more pronounced in populations with chronic ailments such as fibromyalgia, chronic migraine or chronic fatigue syndrome, the duration of the disorder may be an important factor [22, 50]. Additionally, in a study conducted by Lorduy *et al.* [22], a high prevalence of CS was found in the group of participants with comorbidities, while in the present study, comorbidities were not analyzed because we excluded subjects with severe comorbidities. The observed variation between these findings may also be attributed to different sample selection and demographic characteristics of the study populations. Besides, the criteria used to define and measure CS could play an important role, since not all articles use the CSI. This can affect the differences in the results of the present study and other studies. Other methods of analyzing CS include somatosensory assessment, quantitative sensory testing (QSTs), and conditioned pain modulation tests (CPM) [49, 51].

Furthermore, statistical analysis revealed the absence of a direct correlation between specific TMD diagnoses and the level of CS. This is contrary to the findings of La Touche *et al.* [49], who identified a correlation between CS and myogenous TMD. Additionally, the intensity of MMP did not increase with the rise in the severity of CS. These observations are in contrast with expected results based on existing literature and compel a reconsideration of the mechanisms involved in this phenomenon [18, 52]. However, statistical analysis revealed that if we divide the study participants into two categories—those with CS symptoms and those with subclinical level of CS, the subclinical group demonstrates significantly lower masticatory muscle pain. Moreover, the subclinical group presented significantly lower GCPS scores. Marina Jardim *et al.* [53] in their study observed a direct proportional relationship between CS and chronic TMD pain, but it was not associated with pain variables such as pain intensity or the number of painful sites. Baroni *et al.* [23] reported that patients with chronic orofacial pain presented a significant reduction in pressure pain threshold compared to healthy subjects. Ferrillo *et al.* [1] observed that myogenous TMD could manifest as chronic primary pain associated with dysfunction in the CNS as a consequence of CS. The authors of the latter paper conclude that this mechanism may cause increased sensitivity in patients with TMD.

For a better understanding of the pain mechanisms in TMD patients, the work of Adams and Turk regarding the biopsychosocial model provides a more comprehensive view. As mentioned earlier, this model suggests that pain arises from a combination of biological, psychological and social factors, rather than CS alone [17]. This perspective finds support in other research papers focused on pain mechanisms [9, 54, 55]. Additionally, it is important to note that emotional distress can also be connected with CS [23, 54]. Besides that, Reid *et al.* [56] have found that higher pain sensitivity, lower masseter PPT, and increased CS are related to a lower percentage of rapid eye movement (REM) sleep in participants with TMD. This broad spectrum of factors underscores why a direct correlation between specific TMD diagnoses and CS severity may not be apparent. The clinical implications of this information are that patients with CS require a very thorough evaluation of many aspects of their daily lives.

The second tool used in our study was the SSS-8. This questionnaire has been proven useful in assessing the clinical severity of somatization [57]. We evaluated the relationship between the results of this questionnaire, TMD, CS and MMP intensity. Interestingly, this analysis revealed that women reported higher SSS-8 scores, highlighting the sex difference in somatic symptom reporting. These findings align with a wider range of pain-related analyses, which showed that women are more prone to report even mild pain during clinical examinations, whereas men typically only report pain when it is severe [55]. Despite these sex differences, our analysis did not find a direct relationship between SSS-8 scores and specific TMD diagnoses. Once again, we observe the complexity of the causes of TMD, which extend beyond a single type of disease diagnosis, highlighting the multifaceted nature of this condition.

Interestingly, a significant correlation was observed between the results of this SSS-8 and masticatory muscle pain, emphasizing the influence of muscle pain on the perception of somatic symptoms among TMD patients. This relationship indicates that masticatory muscle pain might play an important role in the somatic symptom burden experienced by TMD patients. The literature supports this thesis and suggests that pain in the masticatory muscles may increase the overall symptom burden and is also associated with anxiety and depression [9].

Our findings indicate that individuals with TMD and CS experience higher somatization, as assessed with SSS-8, than those with a subclinical level of CS. Considering the fact that CS is associated with an increased sensitivity of the CNS, this situation may not only lead to an enhanced perception of pain but also seems to provoke a situation in which somatic symptoms are more easily generated and perceived [58]. Similarly, the study by Hashimoto *et al.* [28] highlights how useful the SSS-8 questionnaire is for identifying severe cases of somatic symptoms in patients with CSS. These findings, combined with the correlation of greater intensity of masticatory muscle pain in individuals with higher SSS-8 scores, suggest that somatic symptom severity could serve as a valuable marker for CS. Considering the limited number of studies analyzing somatization based on SSS-8, this area should be further investigated. Addressing CS and somatization simultaneously can lead to more precise and effective treatment strategies.

The limitations of this study include the exclusive use of the CSI for assessing CS and the SSS-8 for somatization assessment and lack of information about previous head and/or neck trauma. The lack of precise analysis of the duration of TMD symptoms and MMP can also be considered a limitation. Additionally, information on non-medication substances that could affect nervous system activity, such as herbal preparations, dietary supplements or energy drinks, was not collected. However, the strength of this study lies in the analysis of a large group of TMD patients without severe comorbidities.

The outcomes of this study supplement the existing body of literature concerning the interactions between temporomandibular disorders, somatic symptoms and CS. Our findings underscore the necessity for further exploration of these phenomena. A key insight from this study is the importance of examining not only specific complaints reported by patients, such as pain in the masticatory muscles, but also the entire range of factors that may influence the occurrence of symptoms, based on the biopsychosocial model.

## 5. Conclusions

This study examined the prevalence of CS and somatization among patients with TMD, revealing that severe CS is less prevalent in this population than previously thought, based on data collected from study participants. Notably, women reported higher somatic symptom burdens than men, emphasizing sex differences in pain experience. Participants with subclinical level of central sensitization demonstrated significantly lower masticatory muscle pain severity in comparison to all other groups combined. A significant correlation was found between the SSS-8 questionnaire and chronic MMP. Furthermore, the subclinical category of the CSI showed significantly lower SSS-8 results than remaining groups combined.

## References

[1] Ferrillo M, Giudice A, Marotta N, Fortunato F, Di Venere D, Ammendolia A, *et al*. Pain management and rehabilitation for central sensitization in temporomandibular disorders: a comprehensive review. International Journal of Molecular Sciences. 2022; 23: 12164. 10.3390/ijms232012164 PMC9602546 36293017

[2] Ramos-Herrada RM, Arriola-Guillén LE, Atoche-Socola KJ, Bellini-Pereira SA, Castillo AA. Effects of botulinum toxin in patients with myofascial pain related to temporomandibular joint disorders: a systematic review. Dental and Medical Problems. 2022; 59: 271–280. 10.17219/dmp/145759 35775414

[3] Valesan LF, Da-Cas CD, Réus JC, Denardin ACS, Garanhani RR, Bonotto D, *et al. *Prevalence of temporomandibular joint disorders: a systematic review and meta-analysis. Clinical Oral Investigations. 2021; 25: 441–453. 10.1007/s00784-020-03710-w 33409693

[4] Wieckiewicz M, Grychowska N, Nahajowski M, Hnitecka S, Kempiak K, Charemska K, *et al*. Prevalence and overlaps of headaches and pain-related temporomandibular disorders among the polish urban population. Journal of Oral & Facial Pain and Headache. 2020; 34: 31–39. 10.11607/ofph.2386 31465030

[5] Schiffman E, Ohrbach R, Truelove E, Look J, Anderson G, Goulet JP, *et al*. Diagnostic criteria for temporomandibular disorders (DC/TMD) for clinical and research applications: recommendations of the international RDC/TMD consortium network and orofacial pain special interest group. Journal of Oral & Facial Pain and Headache. 2014; 28: 6–27. 10.11607/jop.1151 PMC4478082 24482784

[6] Meloto CB, Slade GD, Lichtenwalter RN, Bair E, Rathnayaka N, Diatchenko L, *et al.* Clinical predictors of persistent temporomandibular disorder in people with first-onset temporomandibular disorder. The Journal of the American Dental Association. 2019; 150: 572–581.e10. 10.1016/j.adaj.2019.03.023 31248483

[7] Bueno CH, Pereira DD, Pattussi MP, Grossi PK, Grossi ML. Gender differences in temporomandibular disorders in adult populational studies: a systematic review and meta‐analysis. Journal of Oral Rehabilitation. 2018; 45: 720–729. 10.1111/joor.12661 29851110

[8] Ohrbach R, Dworkin SF. The evolution of TMD diagnosis: past, present, future. Journal of Dental Research. 2016; 95: 1093–1101. 10.1177/0022034516653922 PMC5004241 27313164

[9] Wieckiewicz M, Jenca A Jr, Seweryn P, Orzeszek S, Petrasova A, Grychowska N, *et al.* Determination of pain intensity, pain-related disability, anxiety, depression, and perceived stress in Polish adults with temporomandibular disorders: a prospective cohort study. Frontiers in Integrative Neuroscience. 2022; 16: 1026781. 10.3389/fnint.2022.1026781 PMC9668250 36407294

[10] Seweryn P, Orzeszek SM, Waliszewska-Prosół M, Jenča A, Osiewicz M, Paradowska-Stolarz A,* et al*. Relationship between pain severity, satisfaction with life and the quality of sleep in Polish adults with temporomandibular disorders. Dental and Medical Problems. 2023; 60: 609–617. 10.17219/dmp/171894 37873974

[11] Osiewicz MA, Lobbezoo F, Loster BW, Loster JE, Manfredini D. Frequency of temporomandibular disorders diagnoses based on RDC/TMD in a Polish patient population. CRANIO®. 2018; 36: 304–310. 10.1080/08869634.2017.1361052 28792365

[12] Cigdem Karacay B, Sahbaz T. Investigation of the relationship between probable sleep bruxism, awake bruxism and temporomandibular disorders using the Diagnostic Criteria for Temporomandibular Disorders (DC/TMD). Dental and Medical Problems. 2023; 60: 601–608. 10.17219/dmp/158926 36651343

[13] Fillingim RB, Slade GD, Greenspan JD, Dubner R, Maixner W, Bair E, *et al*. Long-term changes in biopsychosocial characteristics related to temporomandibular disorder: findings from the OPPERA study. Pain. 2018; 159: 2403–2413. 10.1097/j.pain.0000000000001348 PMC6193833 30028791

[14] Yang Y, Xu LL, Liu SS, Lu SJ, Liu LK, Zeng H, *et al.* Analysis of risk factors and interactions for pain in temporomandibular disorder: a cross‐sectional study. Journal of Oral Rehabilitation. 2024; 51: 1113–1122. 10.1111/joor.13682 38486502

[15] Cayrol T, van den Broeke EN, Gerard E, Meeus M, Mouraux A, Roussel N, *et al*. Chronic temporomandibular disorders are associated with higher propensity to develop central sensitization: a case-control study. Pain. 2023; 164: e251–e258. 10.1097/j.pain.0000000000002803 36251966

[16] Harper DE, Schrepf A, Clauw DJ. Pain mechanisms and centralized pain in temporomandibular disorders. Journal of Dental Research. 2016; 95: 1102–1108. 10.1177/0022034516657070 PMC5004242 27422858

[17] Adams LM, Turk DC. Central sensitization and the biopsychosocial approach to understanding pain. Journal of Applied Biobehavioral Research. 2018; 23: e12125.

[18] Campi LB, Jordani PC, Tenan HL, Camparis CM, Gonçalves DA. Painful temporomandibular disorders and central sensitization: implications for management—a pilot study. International Journal of Oral and Maxillofacial Surgery. 2017; 46: 104–110. 10.1016/j.ijom.2016.07.005 27553896

[19] Wang XY, Zhou HR, Wang S, Liu CY, Qin GC, *et al.* NR2B-Tyr phosphorylation regulates synaptic plasticity in central sensitization in a chronic migraine rat model. The Journal of Headache and Pain. 2018; 19: 102. 10.1186/s10194-018-0935-2 PMC6755586 30400767

[20] Li J, Zhang L, Xu C, Lin YH, Zhang Y, Wu HY, *et al.* Prolonged use of NMDAR antagonist develops analgesic tolerance in neuropathic pain via nitric oxide reduction-induced GABAergic disinhibition. Neurotherapeutics. 2020; 17: 1016–1030. 10.1007/s13311-020-00883-w PMC7609518 32632774

[21] Monaco A, Cattaneo R, Marci MC, Pietropaoli D, Ortu E. Central sensitization-based classification for temporomandibular disorders: a pathogenetic hypothesis. Pain Research and Management. 2017; 2017: 5957076. 10.1155/2017/5957076 PMC5592418 28932132

[22] Lorduy KM, Liegey-Dougall A, Haggard R, Sanders CN, Gatchel RJ. The prevalence of comorbid symptoms of central sensitization syndrome among three different groups of temporomandibular disorder patients. Pain Practice. 2013; 13: 604–613. 10.1111/papr.12029 PMC3634891 23336585

[23] Baroni A, Severini G, Straudi S, Buja S, Borsato S, Basaglia N. Hyperalgesia and central sensitization in subjects with chronic orofacial pain: analysis of pain thresholds and EEG biomarkers. Frontiers in Neuroscience. 2020; 14: 552650. 10.3389/fnins.2020.552650 PMC7689025 33281540

[24] Yap AU, Kim S, Lee BM, Jo JH, Park JW. Correlates of jaw functional limitation, somatization and psychological distress among different temporomandibular disorder diagnostic subtypes. Journal of Oral Rehabilitation. 2024; 51: 287–295. 10.1111/joor.13606 37849410

[25] Felin GC, Tagliari CVDC, Agostini BA, Collares K. Prevalence of psychological disorders in patients with temporomandibular disorders: a systematic review and meta-analysis. Journal of Prosthetic Dentistry. 2024; 132: 392–401. 10.1016/j.prosdent.2022.08.002 36114016

[26] Takeuchi T, Hashimoto K, Koyama A, Asakura K, Hashizume M. The Association of central sensitisation with depression, anxiety, and somatic symptoms: a cross-sectional study of a mental health outpatient clinic in Japan. Life. 2024; 14: 612. 10.3390/life14050612 PMC11122528 38792633

[27] Yap AU, Marpaung C. Psychological factors in temporomandibular disorders and somatization: a multidimensional analysis of personality, coping, and distress among young adults. To be published in The International Journal of Prosthodontics. 2023. [Preprint]. 10.11607/ijp.8590 37824118

[28] Hashimoto K, Takeuchi T, Hiiragi M, Koyama A, Nakamura Y, Hashizume M. Utility and optimal cut-off point of the somatic symptom scale-8 for central sensitization syndrome among outpatients with somatic symptoms and related disorders. BioPsychoSocial Medicine. 2022; 16: 24. 10.1186/s13030-022-00253-2 PMC9694559 36434700

[29] Vale Braido GVD, Svensson P, Dos Santos Proença J, Mercante FG, Fernandes G, de Godoi Gonçalves DA. Are central sensitization symptoms and psychosocial alterations interfering in the association between painful TMD, migraine, and headache attributed to TMD? Clinical Oral Investigations. 2023; 27: 681–690. 10.1007/s00784-022-04783-5 36383296

[30] International classification of orofacial pain, 1st edition (ICOP). Cephalalgia. 2020; 40: 129–221. 10.1177/0333102419893823 32103673

[31] International Network for Orofacial Pain and Related Disorders Methodology. Previously known as: international RDC/TMD consortium network. 2017. Available at: https://ubwp.buffalo.edu/rdc-tmdinternational/ (Accessed: December 2017).

[32] International Network for Orofacial Pain and Related Disorders Methodology. Diagnostic Criteria for Temporomandibular Disorders (2014). 2014. Available at: https://ubwp.buffalo.edu/rdc-tmdinternational/tmd-assessmentdiagnosis/dc-tmd/ (Accessed: December 2017).

[33] Peck CC, Goulet JP, Lobbezoo F, Schiffman EL, Alstergren P, Anderson GC, *et al.* Expanding the taxonomy of the diagnostic criteria for temporomandibular disorders. Journal of Oral Rehabilitation. 2014; 41: 2–23. 10.1111/joor.12132 PMC4520529 24443898

[34] Nugent SM, Lovejoy TI, Shull S, Dobscha SK, Morasco BJ. Associations of pain numeric rating scale scores collected during usual care with research administered patient reported pain outcomes. Pain Medicine. 2021; 22: 2235–2241. 10.1093/pm/pnab110 PMC8677438 33749760

[35] Cuesta-Vargas AI, Neblett R, Chiarotto A, Kregel J, Nijs J, van Wilgen CP, *et al.* Dimensionality and reliability of the central sensitization inventory in a pooled multicountry sample. The Journal of Pain. 2018; 19: 317–329. 10.1016/j.jpain.2017.11.006 29198933

[36] Kosińska B, Tarnacka B, Turczyn P, Gromadzka G, Malec-Milewska M, Janikowska-Hołowenko D, *et al.* Psychometric validation of the Polish version of the central sensitization inventory in subjects with chronic spinal pain. BMC Neurology. 2021; 21: 483. 10.1186/s12883-021-02510-3 PMC8665486 34893020

[37] Turczyn P, Kosińska B, Janikowska-Hołoweńko D, Malec-Milewska M, Marszalec N, Maleszka P, *et al*. Translation and cross-cultural adaptation of the Polish central sensitization inventory. Rheumatology. 2019; 57: 129–134. 10.5114/reum.2019.86422 PMC6710845 31462827

[38] Neblett R, Hartzell MM, Cohen H, Mayer TG, Williams M, Choi Y,* et al.* Ability of the central sensitization inventory to identify central sensitivity syndromes in an outpatient chronic pain sample. The Clinical Journal of Pain. 2015; 31: 323–332. 10.1097/AJP.0000000000000113 24806467

[39] Gierk B, Kohlmann S, Kroenke K, Spangenberg L, Zenger M, Brähler E, *et al*. The somatic symptom scale-8 (SSS-8): a brief measure of somatic symptom burden. JAMA Internal Medicine. 2014; 174: 399–407. 10.1001/jamainternmed.2013.12179 24276929

[40] Toussaint A, Hüsing P, Kohlmann S, Löwe B. Detecting DSM-5 somatic symptom disorder: criterion validity of the patient health questionnaire-15 (PHQ-15) and the somatic symptom scale-8 (SSS-8) in combination with the somatic symptom disorder-B criteria scale (SSD-12). Psychological Medicine. 2020; 50: 324–333. 10.1017/S003329171900014X 30729902

[41] Alhalal E, Jackson KT. Evaluation of the Arabic version of the chronic pain grade scale: psychometric properties. Research in Nursing & Health. 2021; 44: 403–412. 10.1002/nur.22116 33586152

[42] Alkhubaizi Q, Khalaf ME, Faridoun A. Prevalence of temporomandibular disorder-related pain among adults seeking dental care: a cross-sectional study. International Journal of Dentistry. 2022; 2022: 3186069. 10.1155/2022/3186069 PMC9467697 36105380

[43] Kimmey BA, McCall NM, Wooldridge LM, Satterthwaite TD, Corder G. Engaging endogenous opioid circuits in pain affective processes. Journal of Neuroscience Research. 2022; 100: 66–98. 10.1002/jnr.24762 PMC8197770 33314372

[44] Bongiovanni T, Gan S, Finlayson E, Ross JS, Harrison JD, Boscardin J, *et al.* Use of muscle relaxants after surgery in traditional medicare part D enrollees. Drugs Aging. 2024; 41: 615–622. 10.1007/s40266-024-01124-x PMC11249446 38980644

[45] Siddiqui TG, Cheng S, Gossop M, Kristoffersen ES, Grambaite R, Lundqvist C. Association between prescribed central nervous system depressant drugs, comorbidity and cognition among hospitalised older patients: a cross-sectional study. BMJ Open. 2020; 10: e038432. 10.1136/bmjopen-2020-038432 PMC7389767 32718926

[46] Bhagavati S. Autoimmune disorders of the nervous system: pathophysiology, clinical features, and therapy. Frontiers in Neurology. 2021; 12: 664664. 10.3389/fneur.2021.664664 PMC8079742 33935958

[47] Pimentel E, Sivalingam K, Doke M, Samikkannu T. Effects of drugs of abuse on the blood-brain barrier: a brief overview. Frontiers in Neuroscience. 2020; 14: 513. 10.3389/fnins.2020.00513 PMC7326150 32670001

[48] Schiffman E, Ohrbach R. Executive summary of the diagnostic criteria for temporomandibular disorders for clinical and research applications. The Journal of the American Dental Association. 2016; 147: 438–445. 10.1016/j.adaj.2016.01.007 PMC4884471 26922248

[49] La Touche R, Paris-Alemany A, Hidalgo-Pérez A, López-de-Uralde-Villanueva I, Angulo-Diaz-Parreño S, Muñoz-García D. Evidence for central sensitization in patients with temporomandibular disorders: a systematic review and meta-analysis of observational studies. Pain Practice. 2018; 18: 388–409. 10.1111/papr.12604 28557358

[50] Ferrari MD, Goadsby PJ, Burstein R, Kurth T, Ayata C, Charles A, *et al*. Migraine. Nature Reviews Disease Primers. 2022; 8: 2. 10.1038/s41572-021-00328-4 35027572

[51] Yücel FN, Duruöz MT. Central sensitization in axial spondyloarthritis: an explorative study with quantitative sensory testing and clinical scales. Modern Rheumatology. 2022; 32: 1137–1145. 10.1093/mr/roab110 34865130

[52] Meng H, Dai J, Li Y. Quantitative sensory testing in patients with the muscle pain subtype of temporomandibular disorder: a systemic review and meta-analysis. Clinical Oral Investigations. 2021; 25: 6547–6559. 10.1007/s00784-021-04171-5 34487241

[53] Jardim ML, Mélo AM, Melchior M. de O, Magri LV. Catastrophizing, central sensitization and chronic pain-related TMD: how is this association? Revista Gestão e Conhecimento. 2022; 16: 565–578.

[54] Ji RR, Nackley A, Huh Y, Terrando N, Maixner W. Neuroinflammation and central sensitization in chronic and widespread pain. Anesthesiology. 2018; 129: 343–366. 10.1097/ALN.0000000000002130 PMC6051899 29462012

[55] Casale R, Atzeni F, Bazzichi L, Beretta G, Costantini E, Sacerdote P, *et al*. Pain in women: a perspective review on a relevant clinical issue that deserves prioritization. Pain and Therapy. 2021; 10: 287–314. 10.1007/s40122-021-00244-1 PMC8119594 33723717

[56] Reid MJ, Hamilton KR, Nilsson SJ, Owens MA, Phillips JL, Finan PH, *et al.* Elevated pain sensitivity is associated with reduced REM sleep in females with comorbid temporomandibular disorder and insomnia. Pain Medicine. 2024; 25: 434–443. 10.1093/pm/pnae022 PMC11224587 38548665

[57] Narrow WE, Clarke DE, Kuramoto SJ, Kraemer HC, Kupfer DJ, Greiner L, *et al*. DSM-5 field trials in the United States and Canada, part III: development and reliability testing of a cross-cutting symptom assessment for DSM-5. American Journal of Psychiatry. 2013; 170: 71–82. 10.1176/appi.ajp.2012.12071000 23111499

[58] Volcheck MM, Graham SM, Fleming KC, Mohabbat AB, Luedtke CA. Central sensitization, chronic pain, and other symptoms: Better understanding, better management. Cleveland Clinic Journal of Medicine. 2023; 90: 245–254. 10.3949/ccjm.90a.22019 37011956

